# Crystal structure of 1,1′-bis­(2-meth­oxy­carbonyl-2-methyl­prop­yl)ferrocene

**DOI:** 10.1107/S2056989015020642

**Published:** 2015-11-07

**Authors:** Yan-Feng Guo, Jian-Jun Wang, Wei-Juan Xu, Dong-Hao Sun, Qiang Gao

**Affiliations:** a College of Chemistry, Chemical Engineering and Materials Science, Soochow University, Suzhou, Jiangsu 215123, People’s Republic of China; b Key Laboratory of Eco-textiles, Ministry of Education, College of Textile & Clothing, Jiangnan University, Wuxi 214122, People’s Republic of China

**Keywords:** crystal structure, inversion symmetry, disubstituted ferrocene, ester

## Abstract

The Fe atom in the title ferrocene derivative, [Fe(C_11_H_15_O_2_)_2_], is situated on an inversion centre. As a result of the point-group symmetry -1 of the mol­ecule, the ferrocene moiety adopts a staggered conformation. The average Fe—C(Cp) bond length (Cp is cyclo­penta­dien­yl) is 2.045 (4) Å, in agreement with that of other disubstituted ferrocenes. The Fe—C bond length involving the substituted C atom is slightly longer [2.0521 (17) Å] than the remaining Fe—C bond lengths caused by the inductive effect of the methyl­ene group on the Cp ring. Apart from van der Waals forces, no significant inter­molecular inter­actions are observed in the crystal packing.

## Related literature   

The inter­est in disubstituted ferrocene compounds has increased due to their applications in the field of homogeneous catalysis, biology and medicine (Atkinson *et al.*, 2004 ▸; Gao *et al.*, 2009 ▸; Ferreira *et al.*, 2006 ▸). The presence of ester groups on these compounds make them promising candidates for the construction of metal-containing polymers (Wilbert *et al.*, 1995 ▸). Related structures have been described by Woodward *et al.* (1952 ▸); Cetina *et al.* (2003 ▸); Navarro *et al.* (2004 ▸); Pérez *et al.* (2015 ▸).
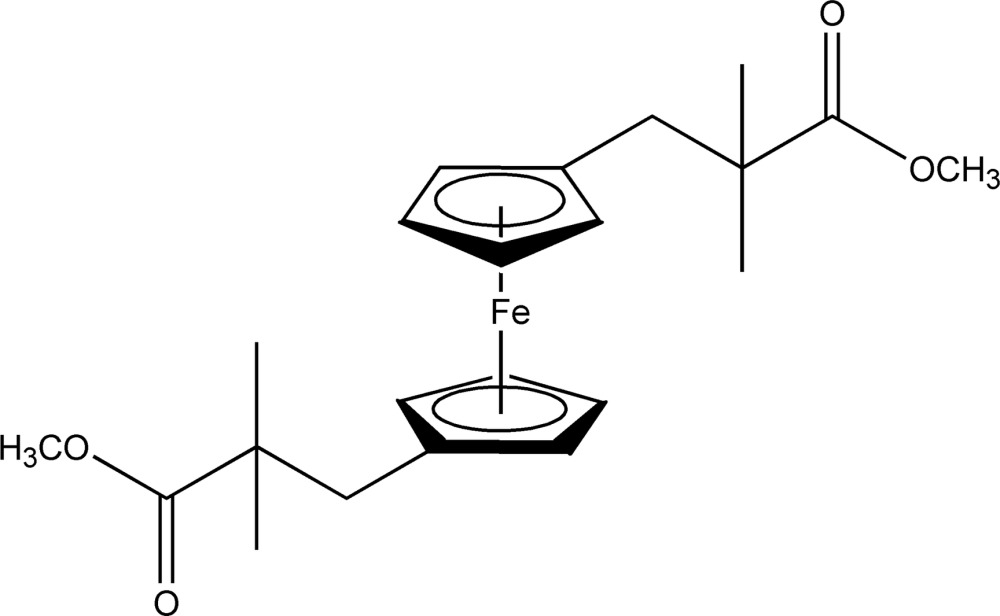


## Experimental   

### Crystal data  

[Fe(C_11_H_15_O_2_)_2_]
*M* *_r_* = 414.31
Triclinic, 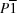

*a* = 6.273 (3) Å
*b* = 8.313 (4) Å
*c* = 10.490 (5) Å
α = 83.833 (6)°
β = 74.405 (7)°
γ = 81.652 (8)°
*V* = 520.0 (4) Å^3^
*Z* = 1
Mo *K*α radiation
μ = 0.75 mm^−1^
*T* = 296 K
0.15 × 0.12 × 0.12 mm

### Data collection  

Bruker APEXII CCD diffractometer
Absorption correction: multi-scan (*SADABS*; Krause *et al.*, 2015 ▸) *T*_min_ = 0.896, *T*_max_ = 0.916
2753 measured reflections
1793 independent reflections
1688 reflections with *I* > 2σ(*I*)
*R*_int_ = 0.013

### Refinement  

*R*[*F* ^2^ > 2σ(*F* ^2^)] = 0.029
*wR*(*F* ^2^) = 0.074
*S* = 1.05
1793 reflections
124 parameters
H-atom parameters constrained
Δρ_max_ = 0.21 e Å^−3^
Δρ_min_ = −0.17 e Å^−3^

### 

Data collection: *APEX2* (Bruker, 2012 ▸); cell refinement: *SAINT* (Bruker, 2012 ▸); data reduction: *SAINT*; program(s) used to solve structure: *SHELXS97* (Sheldrick, 2008 ▸); program(s) used to refine structure: *SHELXL97* (Sheldrick, 2008 ▸); molecular graphics: *DIAMOND* (Brandenburg & Berndt, 1999 ▸); software used to prepare material for publication: *SHELXTL* (Sheldrick, 2008 ▸).

## Supplementary Material

Crystal structure: contains datablock(s) I, New_Global_Publ_Block. DOI: 10.1107/S2056989015020642/wm5231sup1.cif

**Fig. 1.**
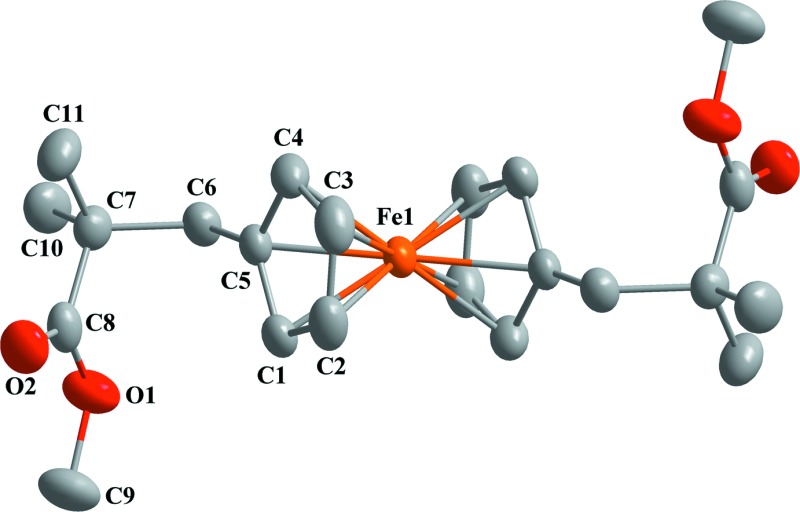
The molecular structure of the title complex, showing displacement ellipsoids drawn at the 50% probability level. All H atoms have been omitted for clarity. Unlabelled atoms are related to labelled ones by the symmetry operation -x, -y + 1, -z + 1.

Structure factors: contains datablock(s) I. DOI: 10.1107/S2056989015020642/wm5231Isup2.hkl

x y z . DOI: 10.1107/S2056989015020642/wm5231fig1.tif
The mol­ecular structure of the title complex, showing displacement ellipsoids drawn at the 50% probability level. All H atoms have been omitted for clarity. Unlabelled atoms are related to labelled ones by the symmetry operation −*x*, −*y* + 1, −*z* + 1.

**Fig. 2.**
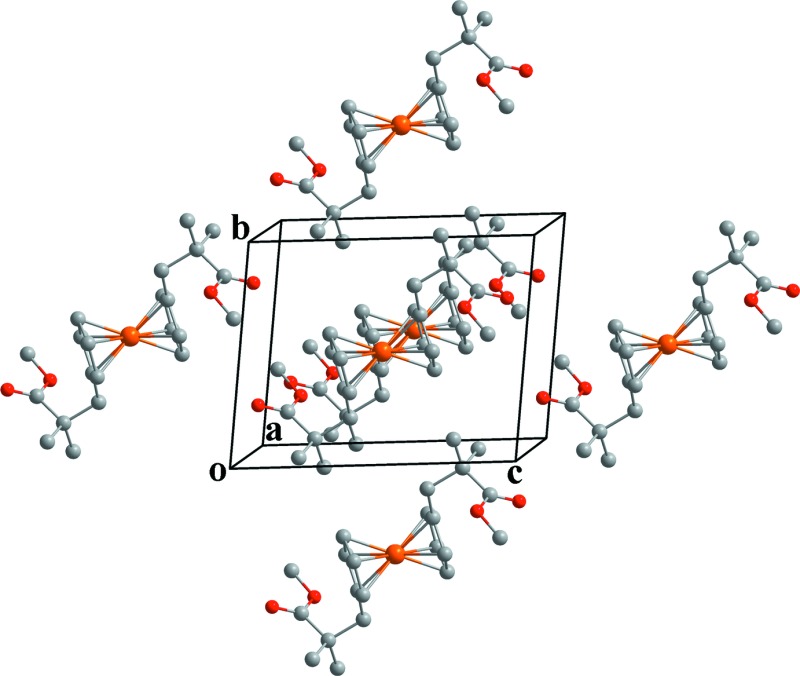
The packing of molecules in the crystal structure of the title compound.

. DOI: 10.1107/S2056989015020642/wm5231fig2.tif
The packing of mol­ecules in the crystal structure of the title compound.

CCDC reference: 1434467

Additional supporting information:  crystallographic information; 3D view; checkCIF report

## supplementary crystallographic information

### S1. Experimental

1,1'-bis(1-methoxy-methyl)ferrocene was first prepared by refluxing 1,1'-bis(hydroxymethyl)ferrocene in methanol and acetic acid (12:1 *v*/*v*) for 16 h. Then a solution of 1,1,-bis(1-methoxy-methyl)ferrocene (3.481 g, 12.7 mmol), 1-methoxy-1-(trimethylsiloxy)-2-methyl-1-propene (10.5 ml, 50.8 mmol) and BF_3_—OEt_2_ (3.5 ml, 27.9 mmol) in CH_2_Cl_2_ (180 ml) was stirred at 195 K for 15 min. The reaction was quenched with a satured solution of NaHCO_3_ and extracted with CH_2_Cl_2_. The organic phases were combined and dried to give a viscous yellow oil, which was chromatographed over a column of silica gel using ethyl acetate/petroleum ether (1:4 *v*/*v*) as the eluent. Yellow crystals of the title compound were obtained by slow evaporation of a solution in dichloromethane/petroleum ether (333-363 K). ^1^H NMR (400 MHz, CDCl_3_) δ 3.97 (d, 8H, C_5_H_4_FeC_5_H_4_), 3.62 (s, 6H, OCH_3_), 2.57 (s, 4H, CH_2_), 1.08 (s, 12H, C(CH_3_)_2_). HRMS (ESI): C_22_H_30_FeO_4_ calcd for [*M* + H]^+^ 415.1572, found 415.1575.

### S2. Refinement

H atoms were placed in calculated positions and thereafter treated as riding atoms, with C—H = 0.98 Å and *U*_iso_(H) = 1.2*U*_eq_(C) (Cp rings CH), 0.97 Å and *U*_iso_(H) = 1.2*U*_eq_(C) (methylene CH_2_) and 0.96 Å *U*_iso_(H) = 1.5*U*_eq_(C) (methyl CH_3_).

### Crystal data

**Table:**

[Fe(C_11_H_15_O_2_)_2_]	*Z* = 1
*M**_r_* = 414.31	*F*(000) = 220
Triclinic, *P*1	*D*_x_ = 1.323 Mg m^−^^3^
Hall symbol: -P 1	Mo *K*α radiation, λ = 0.71073 Å
*a* = 6.273 (3) Å	Cell parameters from 1589 reflections
*b* = 8.313 (4) Å	θ = 3.3–28.2°
*c* = 10.490 (5) Å	µ = 0.75 mm^−^^1^
α = 83.833 (6)°	*T* = 296 K
β = 74.405 (7)°	Block, yellow
γ = 81.652 (8)°	0.15 × 0.12 × 0.12 mm
*V* = 520.0 (4) Å^3^	

### Data collection

**Table:**

Bruker APEXII CCD diffractometer	1793 independent reflections
Radiation source: fine-focus sealed tube	1688 reflections with *I* > 2σ(*I*)
Graphite monochromator	*R*_int_ = 0.013
φ and ω scans	θ_max_ = 25.0°, θ_min_ = 2.0°
Absorption correction: multi-scan (*SADABS*; Krause *et al.*, 2015)	*h* = −7→5
*T*_min_ = 0.896, *T*_max_ = 0.916	*k* = −9→9
2753 measured reflections	*l* = −12→12

### Refinement

**Table:**

Refinement on *F*^2^	Primary atom site location: structure-invariant direct methods
Least-squares matrix: full	Secondary atom site location: difference Fourier map
*R*[*F*^2^ > 2σ(*F*^2^)] = 0.029	Hydrogen site location: inferred from neighbouring sites
*wR*(*F*^2^) = 0.074	H-atom parameters constrained
*S* = 1.05	*w* = 1/[σ^2^(*F*_o_^2^) + (0.038*P*)^2^ + 0.1517*P*] where *P* = (*F*_o_^2^ + 2*F*_c_^2^)/3
1793 reflections	(Δ/σ)_max_ = 0.001
124 parameters	Δρ_max_ = 0.21 e Å^−^^3^
0 restraints	Δρ_min_ = −0.17 e Å^−^^3^

### Special details

**Table:**

Geometry. All e.s.d.'s (except the e.s.d. in the dihedral angle between two l.s. planes) are estimated using the full covariance matrix. The cell e.s.d.'s are taken into account individually in the estimation of e.s.d.'s in distances, angles and torsion angles; correlations between e.s.d.'s in cell parameters are only used when they are defined by crystal symmetry. An approximate (isotropic) treatment of cell e.s.d.'s is used for estimating e.s.d.'s involving l.s. planes.
Refinement. Refinement of *F*^2^ against ALL reflections. The weighted *R*-factor *wR* and goodness of fit *S* are based on *F*^2^, conventional *R*-factors *R* are based on *F*, with *F* set to zero for negative *F*^2^. The threshold expression of *F*^2^ > σ(*F*^2^) is used only for calculating *R*-factors(gt) *etc*. and is not relevant to the choice of reflections for refinement. *R*-factors based on *F*^2^ are statistically about twice as large as those based on *F*, and *R*- factors based on ALL data will be even larger.

### Fractional atomic coordinates and isotropic or equivalent isotropic displacement parameters (Å^2^)

**Table:**

	*x*	*y*	*z*	*U*_iso_*/*U*_eq_	
Fe1	0.0000	0.5000	0.5000	0.03254 (14)	
O1	0.6653 (2)	0.24987 (19)	0.15534 (14)	0.0536 (4)	
O2	0.4288 (3)	0.2353 (2)	0.03404 (15)	0.0646 (4)	
C8	0.4892 (3)	0.1969 (2)	0.13268 (18)	0.0397 (4)	
C5	0.1330 (3)	0.3282 (2)	0.36443 (16)	0.0335 (4)	
C1	0.1796 (3)	0.4864 (2)	0.30665 (17)	0.0387 (4)	
H1A	0.3282	0.5203	0.2685	0.046*	
C6	0.2998 (3)	0.1817 (2)	0.37491 (17)	0.0377 (4)	
H6A	0.2343	0.1094	0.4496	0.045*	
H6B	0.4288	0.2169	0.3931	0.045*	
C4	−0.1038 (3)	0.3335 (2)	0.40734 (18)	0.0410 (4)	
H4A	−0.1861	0.2426	0.4513	0.049*	
C7	0.3785 (3)	0.0848 (2)	0.24845 (18)	0.0372 (4)	
C11	0.1817 (4)	0.0227 (3)	0.2186 (2)	0.0547 (6)	
H11A	0.2329	−0.0363	0.1400	0.082*	
H11B	0.0746	0.1134	0.2049	0.082*	
H11C	0.1134	−0.0482	0.2920	0.082*	
C10	0.5508 (4)	−0.0594 (3)	0.2709 (2)	0.0519 (5)	
H10A	0.6012	−0.1197	0.1930	0.078*	
H10B	0.4836	−0.1293	0.3452	0.078*	
H10C	0.6753	−0.0192	0.2884	0.078*	
C2	−0.0263 (4)	0.5867 (3)	0.31440 (18)	0.0467 (5)	
H2A	−0.0442	0.7018	0.2825	0.056*	
C9	0.7892 (5)	0.3576 (3)	0.0540 (3)	0.0721 (7)	
H9A	0.9106	0.3869	0.0826	0.108*	
H9B	0.6925	0.4542	0.0387	0.108*	
H9C	0.8468	0.3033	−0.0267	0.108*	
C3	−0.1995 (4)	0.4931 (3)	0.3759 (2)	0.0488 (5)	
H3A	−0.3593	0.5316	0.3944	0.059*	

### Atomic displacement parameters (Å^2^)

**Table:**

	*U*^11^	*U*^22^	*U*^33^	*U*^12^	*U*^13^	*U*^23^
Fe1	0.0326 (2)	0.0351 (2)	0.0286 (2)	−0.00167 (15)	−0.00463 (15)	−0.00833 (14)
O1	0.0475 (8)	0.0685 (10)	0.0448 (8)	−0.0180 (7)	−0.0123 (7)	0.0122 (7)
O2	0.0833 (12)	0.0734 (11)	0.0426 (8)	−0.0069 (9)	−0.0298 (8)	0.0029 (8)
C8	0.0437 (11)	0.0398 (10)	0.0332 (10)	0.0076 (8)	−0.0094 (8)	−0.0109 (8)
C5	0.0359 (9)	0.0366 (9)	0.0269 (8)	−0.0039 (7)	−0.0040 (7)	−0.0089 (7)
C1	0.0447 (11)	0.0408 (10)	0.0270 (9)	−0.0049 (8)	−0.0019 (8)	−0.0060 (7)
C6	0.0409 (10)	0.0396 (10)	0.0306 (9)	−0.0009 (8)	−0.0075 (8)	−0.0034 (7)
C4	0.0371 (10)	0.0479 (11)	0.0395 (10)	−0.0079 (8)	−0.0069 (8)	−0.0138 (8)
C7	0.0375 (10)	0.0338 (9)	0.0394 (10)	0.0018 (8)	−0.0098 (8)	−0.0073 (8)
C11	0.0513 (12)	0.0519 (13)	0.0651 (14)	−0.0067 (10)	−0.0150 (11)	−0.0224 (11)
C10	0.0533 (13)	0.0396 (11)	0.0557 (12)	0.0082 (9)	−0.0100 (10)	−0.0017 (9)
C2	0.0610 (13)	0.0430 (11)	0.0338 (10)	0.0073 (10)	−0.0139 (9)	−0.0068 (8)
C9	0.0694 (16)	0.0802 (18)	0.0595 (15)	−0.0259 (14)	−0.0050 (13)	0.0195 (13)
C3	0.0414 (11)	0.0622 (13)	0.0449 (11)	0.0074 (10)	−0.0155 (9)	−0.0199 (10)

### Geometric parameters (Å, º)

**Table:**

Fe1—C2^i^	2.043 (2)	C6—C7	1.554 (3)
Fe1—C2	2.043 (2)	C6—H6A	0.9700
Fe1—C4^i^	2.044 (2)	C6—H6B	0.9700
Fe1—C4	2.044 (2)	C4—C3	1.418 (3)
Fe1—C3^i^	2.044 (2)	C4—H4A	0.9800
Fe1—C3	2.044 (2)	C7—C11	1.522 (3)
Fe1—C1^i^	2.0445 (19)	C7—C10	1.536 (3)
Fe1—C1	2.0445 (19)	C11—H11A	0.9600
Fe1—C5^i^	2.0521 (17)	C11—H11B	0.9600
Fe1—C5	2.0521 (17)	C11—H11C	0.9600
O1—C8	1.333 (3)	C10—H10A	0.9600
O1—C9	1.446 (3)	C10—H10B	0.9600
O2—C8	1.192 (2)	C10—H10C	0.9600
C8—C7	1.522 (3)	C2—C3	1.400 (3)
C5—C1	1.424 (3)	C2—H2A	0.9800
C5—C4	1.428 (3)	C9—H9A	0.9600
C5—C6	1.501 (3)	C9—H9B	0.9600
C1—C2	1.420 (3)	C9—H9C	0.9600
C1—H1A	0.9800	C3—H3A	0.9800
			
C2^i^—Fe1—C2	180.0	C2—C1—C5	108.24 (18)
C2^i^—Fe1—C4^i^	67.98 (9)	C2—C1—Fe1	69.61 (10)
C2—Fe1—C4^i^	112.02 (9)	C5—C1—Fe1	69.95 (10)
C2^i^—Fe1—C4	112.02 (9)	C2—C1—H1A	125.9
C2—Fe1—C4	67.98 (9)	C5—C1—H1A	125.9
C4^i^—Fe1—C4	180.0	Fe1—C1—H1A	125.9
C2^i^—Fe1—C3^i^	40.08 (9)	C5—C6—C7	113.97 (15)
C2—Fe1—C3^i^	139.92 (9)	C5—C6—H6A	108.8
C4^i^—Fe1—C3^i^	40.61 (8)	C7—C6—H6A	108.8
C4—Fe1—C3^i^	139.39 (8)	C5—C6—H6B	108.8
C2^i^—Fe1—C3	139.92 (9)	C7—C6—H6B	108.8
C2—Fe1—C3	40.08 (9)	H6A—C6—H6B	107.7
C4^i^—Fe1—C3	139.39 (8)	C3—C4—C5	108.21 (18)
C4—Fe1—C3	40.61 (8)	C3—C4—Fe1	69.70 (12)
C3^i^—Fe1—C3	180.0	C5—C4—Fe1	69.92 (10)
C2^i^—Fe1—C1^i^	40.65 (8)	C3—C4—H4A	125.9
C2—Fe1—C1^i^	139.35 (8)	C5—C4—H4A	125.9
C4^i^—Fe1—C1^i^	68.18 (8)	Fe1—C4—H4A	125.9
C4—Fe1—C1^i^	111.82 (8)	C11—C7—C8	110.12 (17)
C3^i^—Fe1—C1^i^	68.00 (9)	C11—C7—C10	109.95 (17)
C3—Fe1—C1^i^	112.00 (9)	C8—C7—C10	108.85 (16)
C2^i^—Fe1—C1	139.35 (8)	C11—C7—C6	110.52 (16)
C2—Fe1—C1	40.65 (8)	C8—C7—C6	108.53 (15)
C4^i^—Fe1—C1	111.82 (8)	C10—C7—C6	108.84 (16)
C4—Fe1—C1	68.18 (8)	C7—C11—H11A	109.5
C3^i^—Fe1—C1	112.00 (9)	C7—C11—H11B	109.5
C3—Fe1—C1	68.00 (9)	H11A—C11—H11B	109.5
C1^i^—Fe1—C1	180.0	C7—C11—H11C	109.5
C2^i^—Fe1—C5^i^	68.48 (8)	H11A—C11—H11C	109.5
C2—Fe1—C5^i^	111.52 (8)	H11B—C11—H11C	109.5
C4^i^—Fe1—C5^i^	40.80 (8)	C7—C10—H10A	109.5
C4—Fe1—C5^i^	139.20 (8)	C7—C10—H10B	109.5
C3^i^—Fe1—C5^i^	68.51 (8)	H10A—C10—H10B	109.5
C3—Fe1—C5^i^	111.49 (8)	C7—C10—H10C	109.5
C1^i^—Fe1—C5^i^	40.67 (7)	H10A—C10—H10C	109.5
C1—Fe1—C5^i^	139.33 (7)	H10B—C10—H10C	109.5
C2^i^—Fe1—C5	111.52 (8)	C3—C2—C1	108.31 (18)
C2—Fe1—C5	68.48 (8)	C3—C2—Fe1	69.99 (12)
C4^i^—Fe1—C5	139.20 (8)	C1—C2—Fe1	69.73 (11)
C4—Fe1—C5	40.80 (8)	C3—C2—H2A	125.8
C3^i^—Fe1—C5	111.49 (8)	C1—C2—H2A	125.8
C3—Fe1—C5	68.51 (8)	Fe1—C2—H2A	125.8
C1^i^—Fe1—C5	139.33 (7)	O1—C9—H9A	109.5
C1—Fe1—C5	40.67 (7)	O1—C9—H9B	109.5
C5^i^—Fe1—C5	180.00 (7)	H9A—C9—H9B	109.5
C8—O1—C9	117.84 (18)	O1—C9—H9C	109.5
O2—C8—O1	123.17 (19)	H9A—C9—H9C	109.5
O2—C8—C7	125.6 (2)	H9B—C9—H9C	109.5
O1—C8—C7	111.19 (16)	C2—C3—C4	108.28 (18)
C1—C5—C4	106.96 (17)	C2—C3—Fe1	69.92 (12)
C1—C5—C6	126.84 (17)	C4—C3—Fe1	69.69 (11)
C4—C5—C6	126.17 (17)	C2—C3—H3A	125.9
C1—C5—Fe1	69.38 (10)	C4—C3—H3A	125.9
C4—C5—Fe1	69.28 (10)	Fe1—C3—H3A	125.9
C6—C5—Fe1	127.75 (13)		

Symmetry code: (i) −*x*, −*y*+1, −*z*+1.
