# Supplementary figures and images for: Crystal structure of 1,1′-bis­(2-meth­oxy­carbonyl-2-methyl­prop­yl)ferrocene
Source: Acta Crystallogr E Crystallogr Commun. 2015 Nov 7;71(Pt 12):m213–4. doi: 10.1107/S2056989015020642 (PMC4719839; doi:10.1107/S2056989015020642)

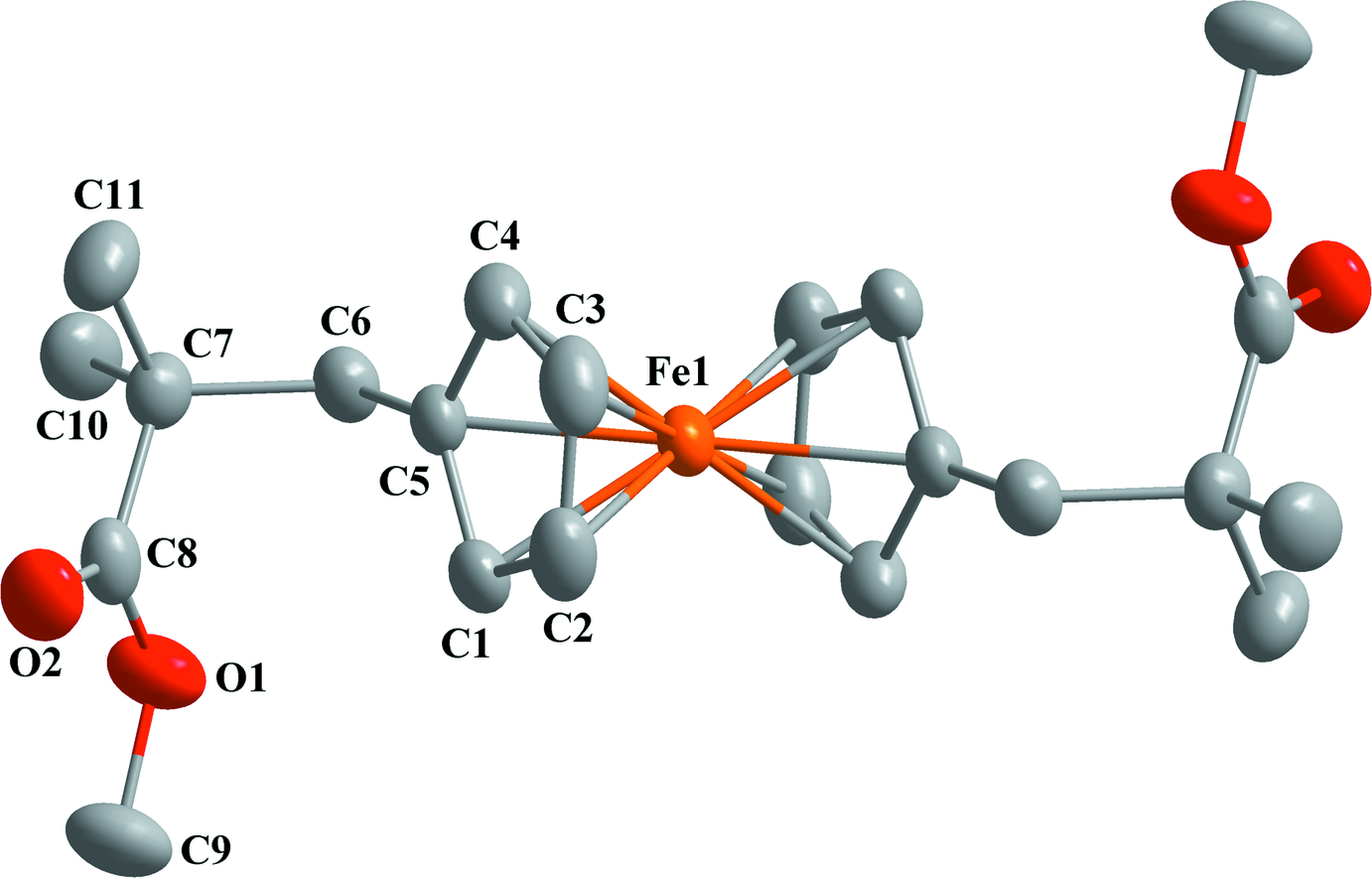

Supplement: Supplementary file 3 [file e-71-0m213-fig1.tif]

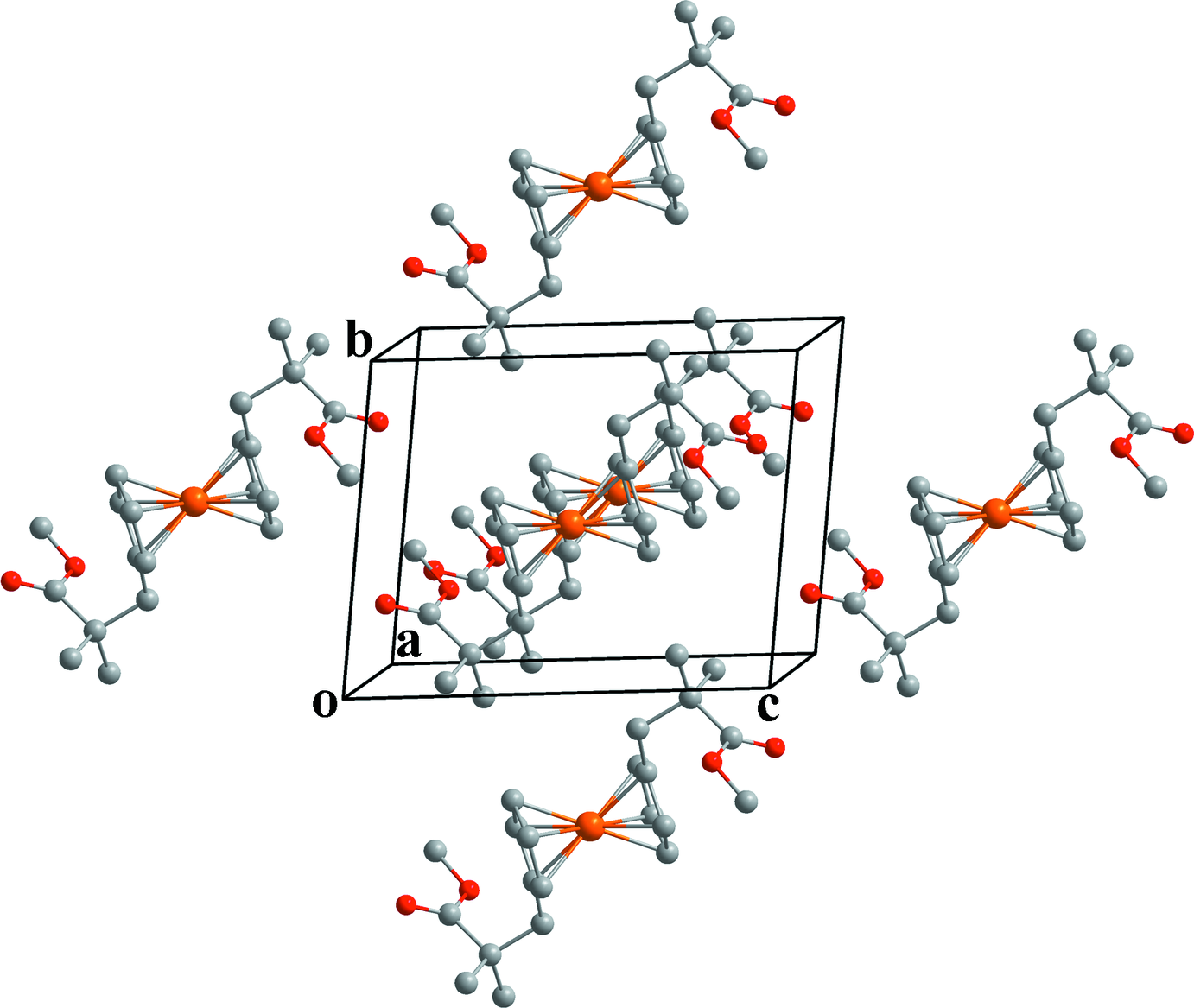

Supplement: Supplementary file 4 [file e-71-0m213-fig2.tif]
